# Real-World Association of SGLT2 Inhibitors with Mortality in Very Elderly Patients with HFrEF and CKD

**DOI:** 10.3390/biomedicines14050980

**Published:** 2026-04-24

**Authors:** Antonio José Bollas Becerra, Marcelino Cortés García, Jorge Balaguer Germán, Carlos Rodríguez-López, José María Romero Otero, José Antonio Esteban Chapel, Luis Nieto Roca, Mikel Taibo Urquía, Ana María Pello Lázaro, José Tuñón

**Affiliations:** 1 Department of Cardiology, Hospital Universitario Fundación Jiménez Díaz, 28040 Madrid, Spain; 2 Faculty of Medicine and Biomedicine, Universidad Alfonso X el Sabio (UAX), 28691 Madrid, Spain; 3 Department of Cardiology, Hospital Universitario Puerta de Hierro, 28222 Majadahonda, Spain; 4 Department of Cardiology, Hospital Universitario Príncipe de Asturias, 28805 Alcala de Henares, Spain; 5 Hospital Universitario Principe de Asturias, Grupo CardioRed1, Carlos III National Institute of Health, 28029 Madrid, Spain; 6 Laboratory of Diabetes and Vascular Pathology, IIS-Fundación Jiménez Díaz, Universidad Autónoma, 28040 Madrid, Spain; 7 Department of Cardiology, Hospital Universitari Son Espases, 07120 Palma de Mallorca, Spain; 8 Department of Medicine, Faculty of Medicine, Universidad Autónoma de Madrid, 28049 Madrid, Spain; 9 Biomedical Research Network on Cardiovascular Diseases CIBERCV, Carlos III National Institute of Health, 28029 Madrid, Spain

**Keywords:** heart failure, sodium-glucose transporter 2 inhibitor, chronic kidney disease, elderly, mortality

## Abstract

**Background**: Heart failure with reduced ejection fraction (HFrEF) and chronic kidney disease (CKD) are common in the growing population of elderly patients, yet little evidence specifically targeting this population exists. The purpose of this study is to analyze the effect of SGLT2 inhibition in this cohort. **Methods**: A single-center, real-world observational study was performed. Patients aged >75 with HFrEF and CKD and theoretical indication for sodium–glucose cotransporter 2 (SGLT2) inhibitors were enrolled. **Results**: A total of 173 patients were included, with a mean age of 84.7 years, mean left ventricle ejection fraction of 29.5% and estimated glomerular filtration rate of 45.9 mL/min/1.73 m^2^. During a median follow-up of 39 months, 73 (42.2%) deaths from any cause and 95 (53.3%) major clinical events (composite of mortality and heart failure admission) were recorded. Multivariate Cox proportional hazards regression analyses were performed to identify associated variables, and SGLT2 inhibition showed to be a protective factor for the mortality endpoint (hazard ratio 0.324 [0.117–0.894]). Male sex was shown to be a risk factor for both endpoints, diabetes mellitus for the mortality endpoint and diuretic use for the major clinical event endpoints. **Conclusions**: In a real-world study, treatment with SGLT2 inhibitors in elderly patients with HFrEF and CKD was associated with a lower rate of all-cause mortality.

## 1. Introduction

Heart failure (HF) is frequent among very elderly patients, with a prevalence of over 20% in patients above 75 years of age [1,2]. In this cohort, heart failure with reduced ejection fraction (HFrEF) represents up to 37–41% of HF [2,3]. Furthermore, HFrEF patients above 75 years of age represent up to 50% of all HFrEF patients [4]. This high prevalence, combined with the progressive aging of society, underscores the importance of effective HFrEF treatment not just in elderly patients, but the population as a whole. Chronic kidney disease (CKD), on the other hand, reaches a prevalence of over 30% in people over 75 years of age [5], standing out as another common clinical condition in very elderly patients.

Sodium–glucose cotransporter 2 inhibitors (SGLT2is), in particular empagliflozin and dapagliflozin, have transcended their initial role as antidiabetic drugs and have become staple medications in the treatment of chronic HFrEF, irrespective of diabetes mellitus status [6,7]. Both European Society of Cardiology (ESC) and American Heart Association (AHA) guidelines recommend their use in HFrEF patients with a class I recommendation [8,9]. Other trials have proven they convey a reduction in the risk of CKD progression and cardiovascular death [10,11].

Research indicates the benefits of SGLT2is in very elderly patients with either HFrEF or CKD, two syndromes that often coexist in the very elderly, yet data on their co-occurrence remains scarce. Existing studies that target elderly patients with HF often aggregate all LVEF categories or do not analyze CKD specifically. A study by Amioka et al. compares SGLT2is and tolvaptan alongside conventional treatment in elderly (≥75 years) HF patients with CKD. This trial shows a reduction in the composite endpoint of HF hospitalizations and cardiac death, but it did not distinguish LVEF categories, thereby including preserved LVEF patients [12]. Similarly, Noiri et al. looked at patients over 80 years of age with HF and SGLT2 inhibition, showing favorable outcomes in patients using SGLT2is, while discontinuation contributed to an unfavorable outcome. However, HFrEF patients comprised only 22% of the cohort, and CKD status was not addressed [13]. Similarly, Silva et al. showed important reductions in a composite of worsening HF and cardiovascular death in patients aged >80 years old with HFrEF treated with SGLT2, with a notable HR of 0.17, yet no stratification by CKD is made [14]. Trials that target elderly CKD patients often do not consider HF. Examples include the real-world study by Kitaoka et al. or the meta-analysis in diabetic kidney disease by Liu et al., showing that SGLT2i slowed eGFR decline, but the study limits findings to CKD and does not consider HF-related endpoints [15,16]. Furthermore, elderly patients have been underrepresented in the main clinical trials for SGLT2i in HFrEF, creating a gap in the evidence base. Data is likewise scarce regarding the effects of SGLT2i on these populations, despite sub-analyses of HFrEF trials showing steady benefit with age [17,18]. Thus, the purpose of this study is to analyze the effect of SGLT2i in very elderly patients with HFrEF and CKD.

## 2. Materials and Methods

We conducted a single-center, real-world, prospective observational study. From November 2019 until November 2022, we consecutively enlisted patients aged 75 years or older with a diagnosis of HFrEF (defined as left ventricular ejection fraction (LVEF) of ≤40% with compatible signs or symptoms), CKD (defined as an estimated glomerular filtration rate (eGFR) of <60 mL/min/1.73 m^2^ as estimated by the Chronic Kidney Disease Epidemiology Collaboration (CKD-EPI) formula) and indication for SGLT2i (regardless of actual prescription status) for diabetes mellitus, CKD or HFrEF. Patients meeting all four criteria were screened from a database compiled in the imaging unit of the Department of Cardiology of University Hospital Fundación Jiménez Díaz (Madrid, Spain). Patients were regularly followed-up, and treatment was optimized by their physician, either a general practitioner or a cardiologist. We reviewed electronic medical records to gather clinical, laboratory, electrocardiographic and echocardiographic data. Echocardiographic exams were performed at the imaging subsection of the Cardiology department of our center. LVEF was assessed either by visual estimation or by Simpson’s biplane method, at the discretion of the sonographer or cardiologist performing the exam and according to standard practice at our center. This study was conducted in accordance with the Declaration of Helsinki and approved by the Clinical Research Ethics Committee of Fundación Jiménez Díaz University Hospital (reference EO093-18 FJD).

Outcomes analyzed in this study were the incidence of all-cause mortality and the first major clinical event, defined as all-cause mortality and the first admission due to HF. Admission due to HF was defined as admission to a healthcare facility lasting more than 24 h due to worsening of HF symptoms, followed by specific treatment for decompensated HF, regardless of the cause of decompensation. Clinical events during follow-up, including death, were collected from the electronic health records of patients or via telephone interviews with patients or their relatives if records were unavailable.

Descriptive statistics are presented as absolute and relative frequencies for qualitative variables and as mean ± standard deviation (SD) for quantitative variables. Univariate analysis for quantitative variables was performed using Student’s *t* test when variables were normally distributed and Mann–Whitney U-test for non-normal distributions. Univariate analysis for qualitative variables was performed using χ^2^ tests or Fisher’s exact test if expected cell frequencies were under 5. The magnitude of the effects of variables was analyzed by means of univariate Cox proportional hazards regression, and results were expressed in the form of hazard ratios (HRs) and 95% confidence intervals (CIs). Multivariate analysis was performed by means of backward stepwise Cox regression. Baseline variables with potential to act as confounding factors were included in the analysis if two criteria were met: first, those with biological and clinical plausibility for confusion; second, if they met the statistical criterion laid out by Mickey and Greenland that suggests excluding variables with a *p* value > 0.20 on univariate analysis [19]. Variables that were selected to be included in the multivariate Cox regression for mortality or first major clinical event were the following qualitative (yes/no) variables: female gender, diabetes mellitus, history of past or current tobacco smoking, obesity, history of past or current atrial fibrillation, history of chronic pulmonary disease, history of stroke, independence in basic activities of daily living, sinus rhythm at admission, New York Heart Association (NYHA) class I or II, improved LVEF, use of SGLT2i, use of beta-blockers, use of angiotensin-converting enzyme inhibitors (ACEIs) or angiotensin II receptor blockers (ARBs) or angiotensin receptor–neprilysin inhibitors (ARNIs), use of digoxin, use of anticoagulant medication, use of digoxin. The quantitative variables were age and eGFR at inclusion. The magnitude of effect of the variables in the multivariate analyses is expressed as HR and 95% CI. Statistical analyses were performed with SPSS 22.0 (SPSS Inc., Chicago, IL, USA).

## 3. Results

### Baseline Characteristics of the Study Population

A total of 173 patients were included in the analysis. Baseline characteristics of the total study population, categorized by use of SGLT2i at the end of follow-up, are presented in Table 1. Table 2 presents treatment at end of follow-up. The mean age was 84.7 years (±5.0) and 33.5% of patients were female. The mean eGFR was 45.9 mL/min/1.73 m^2^ (±11.2), with 40.5% of patients having an eGFR of less than 45 mL/min/1.73 m^2^. The Mean LVEF was 29.5% (±8.0%) and the main cause of systolic dysfunction was ischemic factors (49.1%). The baseline characteristics and treatment at end of follow-up were well balanced between groups, except that SGLT2i users were, on average, 3.4 years younger and used more ACE-I, ARB or ARNI.

After a median follow-up of 39 months, all-cause mortality happened in 73 patients (42.2%), including 4 (21.1%) in the SGLT2i group and 69 (44.8%) in the non-SGLT2i group. A total of 95 patients (54.9%) had a major clinical event, of which 13 (68.4%) were in the SGLT2i group and 82 (53.3%) were in the non-SGLT2i group. A total of 14 patients died of non-sudden cardiac deaths (19.2% of deaths), 40 patients died of non-cardiac causes (54.8% of deaths), 2 patients experienced sudden death, and the cause of death was undetermined in 17 (23.3%) patients.

Because of the observational nature of the study and the differences between subgroups, we designed a two-stage analysis to evaluate the relationship between SGLT2i use and the endpoints of all-cause mortality and the first major clinical event. First, potentially statistically significant covariates were identified using univariate Cox analysis. Then, survival analysis by means of multivariate Cox regression was performed to confirm these variables and exclude potential confounding factors. Table 3 and Table 4 show the results of significant covariates in univariate and multivariate analysis of all-cause mortality and first major clinical event, respectively.

**Figure 1:**
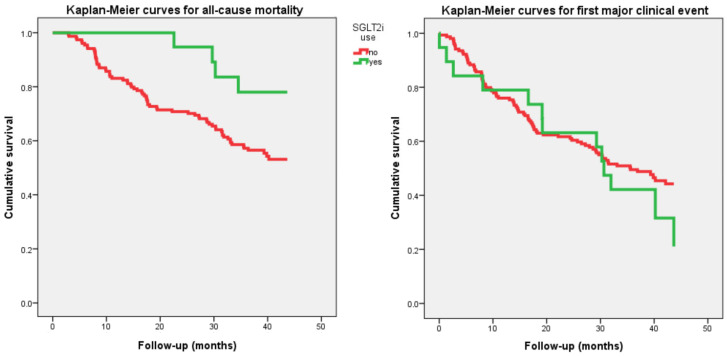
Kaplan–Meier survival curves showing cumulative probability of all-cause mortality (**left**) and first major clinical event (**right**) in patients aged 75 years or older with heart failure with reduced ejection fraction and chronic kidney disease, by SGLT2 inhibitor (SGLT2i) treatment.

Male sex and diabetes mellitus were significantly associated with increased mortality while SGLT2i use was significantly associated with reduced mortality. Male sex and diuretic use, but not SGLT2 inhibition, were significantly associated with the incidence of a first major clinical event. Kaplan–Meier curves for all-cause mortality and first major clinical event, comparing the population treated with an SGLT2i to those not treated with this drug, are shown in Figure 1. As expected from the Cox analyses, the curves diverge significantly in the case of all-cause mortality, but not for the first major clinical event.

Due to the unexpectedly low use of SGLT2is in our cohort, of just 11% of total patients with a theoretical HF indication for these drugs, we decided to investigate reasons for non-utilization of this pharmacological class in our population. By means of electronic health records, we were able to identify the cause in 17 patients not taking SGLT2is (11%). Of these, the main reason given was CKD (13 patients), but only 2 of these patients had an eGFR that contraindicated SGLT2is according to contemporary guidelines. Of the patients in our population, 89% had no clear contraindication for SGLT2is stated in their records.

## 4. Discussion

Sodium–glucose cotransporters (SGLTs) are transporters responsible for the secondary active transport of sodium and glucose, driven by the activity of sodium/potassium-ATPase. Six isoforms have been identified in humans, yet the most clinically relevant isoforms are 1 and 2 [20]. SGLT1 is mainly responsible for glucose absorption in the small intestine, but it is also found in the S2 and S3 segments of the proximal tubule of the nephron, where it is responsible for the uptake of about 3% of filtered glucose [20,21]. SGLT2, on the other hand, is primarily found in the S1 and S2 segments of the proximal tubule, where it is responsible for reabsorbing 97% of filtered glucose. Notably, SGLT1 uptakes two sodium ions for each glucose molecule, while SGLT2 only uptakes one [21,22]. SGLT2 blockade in the kidney is responsible for osmotic diuresis and natriuresis, which is quickly compensated for by more distal segments of the tubule, including an increase in the activity of SGLT1, as well as an increase in the levels of vasopressin. This compensation is believed to limit the risk of severe volume and electrolyte depletion by SGLT2 use [21,23]. Further diuretic effects may come from a reduction in the proximal tubular absorption of chloride. When SGLT2 is inhibited, the tonicity of the tubular fluid increases. Since the proximal tubule is permeable to water, absorption of ions takes place to maintain isotonicity of the fluid and the proximal tubular interstitial fluid: this includes sodium ions and chloride ions. Absorption of chloride ions results in their decreased delivery to the loop of Henle, reducing the action of the sodium–potassium–chloride transporter (Na-K-2Cl), the main driver of loop-of-Henle tubular reabsorption, acting in a similar way to loop diuretics [24].

The plant glycoside phlorizin was first isolated from the bark of the apple tree in 1835. In 1866, the glucosuric and plasma-glucose-lowering effects were discovered, but it would not be until 1933 that the tubular blockade mechanism would be discovered [25,26]. In 1987, Rossetti et al. prove that phlorizin treatment in diabetic rats could normalize tissue sensitivity to insulin [27], opening the door for treatment in adults, yet phlorizin was not apt for this use, since intestinal SGLT1 inhibition caused diarrhea [22]. The focus shifted to SGLT2 inhibition, and in November 2012, the European Medicines Agency approved dapagliflozin, the first of the “gliflozins”, for the treatment of type 2 diabetes mellitus [28]. In the following years, this pharmacological class, and in particular dapagliflozin and empagliflozin, have proven their effectiveness for the prevention of cardiovascular and renal events in patients with diabetes mellitus [29,30], CKD [10,11], HF across all LVEF categories [6,7,31,32], acute myocardial infarction [33,34], acute HF [35] and even patients with aortic stenosis undergoing transcatheter aortic valve implantation [36]. The abundance of evidence for these drugs has led to class I recommendations in ESC Guidelines and AHA for HF management [8,9,37], ESC Guidelines for the management of cardiovascular disease in diabetic patients [38], and Kidney Disease: Improving Global Outcomes (KDIGO) guidelines for CKD [39].

Apart from the previously explained diuretic and glycosuric mechanisms, SGLT2is appear to have off-target effects on the myocardium. This class of drugs may improve cardiac function by suppressing inflammation, oxidative stress, and ion handling in the myocardial cell. Further beneficial mechanisms include optimization of metabolism in the cell, specifically by increasing ketone metabolism and modulating mitochondrial metabolism [40]. Indirect effects on the heart include reduction in vascular stiffness and increased production of erythropoietin, apart from improved renal function [41]. These effects combine to improve cardiac structure, reducing fibrosis, remodeling and hypertrophy [42]. Hyperfiltration and increased glomerular pressure are mechanisms responsible for glomerular damage in CKD. SGLT2is reduce both of these effects, alleviating the stress on the kidneys. Glycosuria appears to increase glucagon secretion, which vasodilates renal blood vessels; it also reduces insulin, responsible for the deleterious expansion of the extracellular matrix [43].

Despite the ample favorable evidence for SGLT2is in HFrEF and CKD, there is considerably less data available for elderly patients with these conditions. DAPA-HF was a placebo-controlled clinical trial on HFrEF patients with class II-IV NYHA that evaluated the effect of adding dapagliflozin alongside recommended therapy. The primary endpoint was a composite of worsening HF (defined as hospitalization or intravenous therapy) or cardiovascular death [6]. EMPEROR-Reduced was an analogous trial with a similar population and similar outcomes but investigated the effect of empagliflozin [7]. Both trials, successful in showing an approximate 25% reduction in the composite endpoints, included around patients of which 25% were above 75 years of age; analyses in these groups have shown consistent benefits of SGLT2 inhibition in this age group compared to other patients [17,18], yet both trials underrepresent true clinical practice, where the proportion of HFrEF patients aged ≥75 years is estimated to be 50% [4]. A few smaller studies have deliberately targeted this population [44,45], also showing benefits in all-cause mortality and cardiovascular events, yet neither performed specific subgroup analyses on patients with CKD.

With regard to the role of the main HF-related SGLT2i in CKD, two large trials must be highlighted. EMPA-KIDNEY was a placebo-controlled clinical trial evaluating the effect of treatment with empagliflozin in patients with an eGFR of 20–45 mL/min/1.73 m^2^ or 45–90 mL/min/1.73 m^2^ with an urinary albumin-to-creatinine ratio of >200 mg/g [11]. DAPA-CKD was a similar trial but with dapagliflozin and recruited patients with an eGFR of 25–75 mL/min/1.73 m^2^ and an urinary albumin-to-creatinine ratio of 200–5000 mg/g [10]. Amongst their outcomes, both trials evaluated progression of CKD as measured by a sustained increase in eGFR, end-stage kidney disease or death from renal causes; they also evaluated death from cardiovascular causes. A prespecified sub-analysis of EMPA-KIDNEY and a separate sub-analysis of DAPA-CKD show a consistent effect of SGLT2is across different age groups, yet very elderly patients remain underrepresented, as the mean age of the population is just 63.9 and 61.8 years and the percentage of patients above 70 years of age is around 30% in both trials [10,11,46], while the percentage of CKD patients above 75 years of age appears to be closer to 60% [47]. Thus, the population presented in the main SGLT2i trials for HFrEF and CKD is not wholly representative of usual clinical care, where elderly patients are the majority. An in-depth search of the literature outputs no studies where the main population combines both advanced age and CKD, resulting in a gap in specific evidence in this HFrEF subgroup, which seems to be a large percentage of total HFrEF patients.

In this real-world, prospective observational study we analyzed the outcomes associated with SGLT2is in this underrepresented cohort of very elderly patients with both HFrEF and CKD. SGLT2i use appeared to be associated with reduced all-cause mortality with borderline statistical significance (*p* = 0.05) after a median follow-up of 39 months. Follow-up was longer than in the landmark trials for empagliflozin and dapagliflozin in CKD and HFrEF (ranging from 16 to 28 months). We consider our result hypothesis-generating due to the borderline significance, yet of clinical relevance as it shows the potential benefits of SGLT2 inhibition with regard to reducing all-cause mortality in this cohort. Elderly patients represent a very large population with less evidence to guide medical treatment and, from our point of view, a low chance of having future randomized trials directed at them because of their high proportion of frailty and comorbidity. Our findings, aimed at a non-selected population, may help guide therapy for a large percentage of HFrEF and CKD patients. Notably, no significant differences were found for the endpoint of first major clinical event, which combines all-cause mortality and hospitalization. These findings contrast with major trials for SGLT2is, where benefit stemmed mainly from reductions in hospitalizations for HF. One possible explanation might be that of competing events: patients that do not die are still susceptible to HF hospitalization, a common event in this highly vulnerable population. Thus, despite preventing death, SGLT2i treatment still contributes to the first major clinical event endpoint by allowing more admissions because of survival and extended follow-up. The chosen cohort, with a high level of comorbidities, is also more susceptible to death from non-cardiovascular causes than the younger and less-comorbid populations used in the landmark trials for these drugs. This higher risk of mortality may be the driver for the signal towards mortality benefit in our study that may be less significant in pivotal trials. An additional factor that could contribute to these differences is that the population chosen for our study is a population that doubly benefits from SGLT2is; landmark trials have shown mortality benefits in both HFrEF [6,18] and CKD [7,10,11], if only as a secondary prespecified endpoint in many of these.

The borderline statistical significance of the difference in the mortality endpoint, and the discrepancy with major trials where benefit was more shifted towards HF hospitalization, may be explained in part by low statistical power. The SGLT2i-using cohort only includes 11% of patients and thus has reduced statistical power, despite a theoretical indication in all patients. When recruitment started in our study, DAPA-HF, showing the benefits of dapagliflozin in HFrEF, had already been published [6] and would be followed shortly after by empagliflozin in the EMPEROR-Reduced trial [7]. Furthermore, the ESC 2019 Guidelines on diabetes and cardiovascular diseases already supported, with a class I indication, the use of SGLT2 inhibition in diabetic patients in three situations [48]. First, in all diabetic patients to reduce risk of HF hospitalization. Second, in diabetics with high cardiovascular risk and/or established cardiovascular disease to reduce risk of cardiovascular events and death. Finally, in diabetic patients with an eGFR from 30 to 89 mL/min/1.73 m^2^ to reduce renal endpoints [48]. Of our patients, 34.7% were diabetic and therefore potential candidates for SGLT2 inhibition for the reduction of the risk of HF hospitalization and reduction in renal endpoints, meaning that at least one in every four patients with a clear guideline-based recommendation in 2019 for these drugs had not been prescribed. Furthermore, once indication for SGLT2is was established, using a limit of eGFR of 30 mL/min/1.73 m^2^, most of our cohort (92%) would have been suitable. One of the possible explanations for this striking underuse of SGLT2 inhibition is that recruitment started before the inclusion of these drugs in the ESC Guidelines on the management of HF, which would include SGLT2is in the 2021 version [8], published after almost 2 years of recruitment.

There are other possible causes behind this low use of SGLT2is. Low utilization may be driven by a fear of side effects in this population, where polypharmacy, comorbidities and frailty are common. SGLT2is are indicated in CKD up to 20 mL/min/1.73 m^2^ and are not only considered safe for kidney function but also “nephroprotective” drugs that slow the decline in kidney function [49]. A common phenomenon when starting these drugs is a “dip” in kidney function, related to hemodynamic effects. By inhibiting sodium reabsorption in the proximal tubule, an increase in sodium delivery to the macula densa produces afferent arteriolar vasodilation and a reduction in intraglomerular pressure and, ultimately, in GFR. This reduction, which can often reach acute kidney injury levels, usually recovers after a period of 12 weeks, and does not preclude the long-term nephroprotective effect of this class of medications [50]. Unfamiliarity with this transient effect may lead clinicians to discontinue these drugs, especially in the context of the other pharmacological classes often used in HFrEF, especially ACEI, ARB and MRA, which have a real potential for worsening kidney function. As older patients have lower eGFR and are more prone to dehydration, clinicians might be more prone to withdrawal of SGLT2is for fear of excessive diuresis and acute kidney injury. However, Hacil et al. show that in a very elderly cohort (mean age 90 years) patients taking SGLT2is had similar rates of acute kidney injury when compared to placebo [44]. Another concern might be the risk of genitourinary infections, which is increased in users of SGLT2is, yet studies suggest no increased risk in elderly patients taking these drugs compared to younger patients, including the safety outcomes of hypotension, acute renal failure, hypoglycemia, constipation and bone fractures; all of which are common comorbidities in elderly patients that might discourage use of SGLT2is [44,51]. Genitourinary infections did not lead to a high rate of discontinuation or adverse outcomes in a large real-world study of SGLT2is in geriatric patients [44]. In our cohort of very elderly patients (mean age 85 years), a borderline significant reduction in mortality was shown. Thus, these results, supported by the literature emphasizing effectiveness and safety, should encourage clinicians to use SGLT2is in very elderly patients with HFrEF and CKD and to carefully consider the benefit–risk balance when withholding these mortality-reducing drugs in case of non-severe and transient adverse events such as genitourinary infections or eGFR dips.

This study has several limitations. The use of a database to identify patients and reliance on healthcare records restricts access to potentially relevant variables, such as frailty indices or albuminuria. The missing data also frequently included the cause of non-use of SGLT2is and the cause of death, which was unavailable in over 23% of patients. Reliance on health records is a major factor in these missing values, since many patients have died outside of our institution and access to outside reports is limited or records may be closed after death without including this information. We aimed to curtail this limitation by using all-cause mortality, making etiology irrelevant for the primary endpoint at the expense of losing information on actual cardiovascular impact. Another major limitation that stems from unavailable data is that time-dependent variables cannot be used during the Cox modeling process. This restriction subjects our study to immortal time bias, since patients in the SGLT2i group may not have been taking the medication during part of the follow-up period, biasing the HR in favor of SGLT2is. The modest sample size, particularly in the SGLT2i group, stems from the real-world nature of the study. At the time of study, SGLT2i utilization was low, and this impacted the treatment arm. This imbalance between groups may have reduced the statistical power of the study, leading only to borderline significance for the mortality endpoint, which must be taken as hypothesis-generating, especially in the context of an observational trial. Other limitations that are inherent to single-center observational studies such as the one presented are those of external validity. Our trial population corresponds to patients directed to Cardiology or the Heart Failure unit of our hospital. Derivation criteria and patterns thus influence the population that we have studied and it may not fully correspond with the general elderly HFrEF and CKD population: there is a chance that these specialized units may treat only more complex and/or less frail patients, where treatment is perceived to have a better chance of improving survival. These limitations lead to results being merely hypothesis-generating, warranting further studies to better define this signal towards benefit.

## 5. Conclusions

Use of SGLT2is was associated with a borderline significant reduction in all-cause mortality in very elderly patients with HFrEF and CKD. Underutilization of these drugs in this cohort of patients was very high despite data showing safety in very elderly patients. Adherence to current clinical guidelines for HF and CKD in these patients could improve their prognosis and use of SGLT2is, a pharmacological class shown to be safe and well-tolerated in this cohort, may play a leading role in individualized treatment.

## Figures and Tables

**Table 1:** Baseline characteristics of the study population.

Variables	Total Population (*n* = 173)	Non-SGLT2i Users (*n* = 154, 89%)	SGLT2i Users (*n* = 19, 11%)	*p*-Value ^a^
**Age, years (SD)**	84.7 (5.0)	85.1 (4.9)	81.7 (4.7)	0.009
**Female,** *n* **(%)**	58 (33.5%)	53 (34.4%)	5 (26.3%)	NS
**Arterial hypertension,** *n* **(%)**	148 (85.5%)	130 (84.4%)	18 (94.7%)	NS
**Diabetes mellitus,** *n* **(%)**	60 (34.7%)	50 (32.5%)	10 (52.6%)	NS
**Dyslipidemia,** *n* **(%)**	98 (56.6%)	86 (55.8%)	12 (63.2%)	NS
**Obesity,** *n* **(%)**	22 (12.7%)	20 (13.0%)	2 (10.5%)	NS
**Chronic obstructive pulmonary disease,** *n* **(%)**	21 (12.1%)	20 (13%)	1 (5.3%)	NS
**Stroke,** *n* **(%)**	26 (15.0%)	24 (15.6%)	2 (10.5%)	NS
**Peripheral artery disease,** *n* **(%)**	20 (11.6%)	17 (11.0%)	3 (15.8%)	NS
**Ischemic etiology of left ventricular systolic dysfunction,** *n* **(%)**	85 (49.1%)	70 (45.5%)	15 (79.0%)	NS
**Previous admissions for HF,** *n* **(%)**	79 (45.7%)	72 (46.8%)	7 (36.8%)	NS
**NYHA class**				
I–II	144 (83.3%)	134 (87.0%)	10 (52.7%)	NS
III–IV	23 (13.2%)	15 (9.7%)	8 (42.0%)	NS
Unknown	6 (3.5%)	5 (3.3%)	1 (5.3%)	NS
**eGFR, mL/min/1.73 m^2^ (SD)**	45.9 (11.2)	45.9 (11.4)	46.2 (9.7)	NS
**Categories of eGFR**				
eGFR 45–59 mL/min/1.73 m^2^, *n* (%)	103 (59.5%)	93 (60.4%)	10 (52.6%)	NS
eGFR 30–44 mL/min/1.73 m^2^, *n* (%)	56 (32.4%)	48 (31.2%)	8 (42.1%)	NS
eGFR 20–29 mL/min/1.73 m^2^, *n* (%)	7 (4.0%)	6 (3.9%)	1 (5.26%)	NS
eGFR < 20 mL/min/1.73 m^2^, *n* (%)	7 (4.0%)	7 (4.6%)	0	NS
**Heart rate, lpm (SD)**	75.5 (16.8)	75.4 (17.3)	76.8 (12.3)	NS
**Sinus rhythm,** *n* **(%)**	101 (58.4%)	88 (57.1%)	10 (68.4%)	NS
**QRS interval duration, ms (SD)**	133.1 (36.2)	133.3 (37.1)	131.5 (29.3)	NS
**LVEF,** *n* **(%)**	29.5 (8.0)	30.0 (7.7)	25.8 (9.2)	NS

Summary statistics of quantitative variables are presented as mean (standard deviation); summary statistics of qualitative variables are presented as number of patients (percentage). eGFR: Estimated glomerular filtration rate, HF: heart failure, LVEF: left ventricular ejection rate, NYHA: New York Heart Association, SD: standard deviation. ^a^ Comparisons between subgroups (analysis of variance for quantitative variables, χ^2^ test for qualitative variables), where NS: not (statistically) significant.

**Table 2:** Treatment of the population at the end of follow-up.

Variables	Total Population (*n* = 173)	Non-SGLT2i Users (*n* = 154, 89%)	SGLT2i Users (*n* = 19, 11%)	*p*-Value ^a^
**Beta-blocker,** *n* **(%)**	139 (80.3%)	121 (78.6%)	18 (94.7%)	NS
**Any of ACEI, ARB, ARNI,** *n* **(%)**	125 (72.3%)	106 (68.8%)	19 (100%)	0.018
**MRA,** *n* **(%)**	65 (37.6%)	55 (35.7%)	10 (52.6%)	NS
**SGLT2 inhibitor,** *n* **(%)**	19 (11%)	0	19 (100%)	--
**Diuretic,** *n* **(%)**	128 (74%)	114 (74.0%)	14 (73.7%)	NS
**Nitrate,** *n* **(%)**	10 (5.8%)	19 (6.5%)	0	NS
**Antiplatelet therapy,** *n* **(%)**	55 (31.8%)	44 (28.6%)	11 (57.9%)	0.010
**Anticoagulant therapy,** *n* **(%)**	106 (61.3%)	97 (63.0%)	9 (47.4%)	NS
**Ivabradine,** *n* **(%)**	4 (2.3%)	3 (2.0%)	1 (5.3%)	NS
**Digoxin,** *n* **(%)**	12 (6.9%)	12 (7.8%)	0	NS
**Amiodarone,** *n* **(%)**	24 (13.9%)	22 (14.3%)	2 (10.5%)	NS
**Cardiac resynchronization therapy,** *n* **(%)**	27 (15.6%)	23 (14.9%)	4 (21.1%)	NS

Summary statistics are presented as number of patients (percentage). ACEI: Angiotensin-converting enzyme inhibitor, ARB: angiotensin receptor blocker, ARNI: angiotensin receptor/neprilysin inhibitor, MRA: mineralocorticoid receptor antagonist, SGLT2: sodium–glucose cotransporter 2. ^a^ Comparisons between subgroups with χ^2^ test for qualitative variables, where NS: not (statistically) significant.

**Table 3:** Univariate and multivariate Cox proportional hazards regression for all-cause mortality.

Variables	Univariate Cox Analysis	Multivariate Cox Analysis
**Age, by year**	HR 1.022 (0.973–1.073)	Excluded
**Male sex**	**HR 2.180 (1.252–3.798)**	**HR 2.279 (1.306–3.978)**
**eGFR, by 1 mL/min/1.73 m^2^**	HR 1.002 (0.982–1.023)	Excluded
**Diabetes mellitus**	HR 1.458 (0.912–2.331)	**HR 1.628 (1.010–2.624)**
**Stroke and/or transient ischemic attack**	HR 1.341 (0.721–2.492)	Excluded
**Chronic pulmonary obstructive disease**	HR 1.122 (0.558–2.254)	Excluded
**Non-sinus rhythm**	**HR 1.712 (1.080–2.713)**	Excluded
**Improvement in LVEF**	HR 0.628 (0.375–1.051)	Excluded
**SGLT2 inhibitors**	**HR 0.380 (0.139–1.041)**	**HR 0.324 (0.117–0.894)**
**Beta-blockers**	HR 0.773 (0.443–1.346)	Excluded
**ACEI, ARB or ARNI**	HR 0.800 (0.504–1.268)	Excluded
**Sacubitril/valsartan**	HR 0.665 (0.371–1.192)	Excluded

ACEI: Angiotensin-converting enzyme inhibitor, ARB: angiotensin receptor blocker, ARNI: angiotensin receptor/neprilysin inhibitor, eGFR: estimated glomerular filtration rate. LVEF: left ventricular ejection fraction. **Bold** values denote statistically significant associations with *p*-values < 0.05.

**Table 4:** Univariate and multivariate Cox proportional hazards regression for first major clinical event.

Variables	Univariate Cox analysis	Multivariate Cox Analysis
**Male sex**	HR 1.542 (0.976–2.436)	**HR 1.763 (1.087–2.860)**
**Obesity**	HR 1.672 (0.962–2.906)	Excluded
**eGFR, by 1 mL/min/1.73 m^2^**	HR 1.002 (0.982–1.023)	Excluded
**Non-sinus rhythm**	HR 1.437 (0.983–2.202)	Excluded
**SGLT2 inhibitors**	HR 1.293 (0.720–2.322)	Excluded
**ACEI, ARB or ARNI**	**HR 0.547 (0.362–0.826)**	Excluded
**Sacubitril/valsartan**	**HR 1.568 (1.013–2.428)**	Excluded
**Diuretics**	**HR 1.646 (1.005–2.697)**	**HR 1.824 (1.074–3.098)**

ACEI: Angiotensin-converting enzyme inhibitor, ARB: angiotensin receptor blocker, ARNI: angiotensin receptor/neprilysin inhibitor, SGLT2: sodium–glucose cotransporter 2. **Bold** values denote statistically significant associations with *p*-values < 0.05.

## Data Availability

The data presented in this study is available on request from the corresponding author.

## Abbreviations

The following abbreviations are used in this manuscript:

ACEI	Angiotensin-converting enzyme inhibitor
AHA	American Heart Association
ARB	Angiotensin II receptor blocker
ARNI	Angiotensin receptor–neprilysin inhibitor
CI	Confidence interval
CKD	Chronic kidney disease
CKD-EPI	Chronic Kidney Disease Epidemiology Collaboration
eGFR	Estimated glomerular filtration rate
ESC	European Society of Cardiology
HF	Heart failure
HFrEF	Heart failure with reduced ejection fraction
HR	Hazard ratio
KDIGO	Kidney Disease Improving Global Outcomes
LVEF	Left ventricular ejection fraction
MRA	Mineralocorticoid receptor antagonist
NYHA	New York Heart Association
SD	Standard deviation
SGLT	Sodium–glucose cotransporter
SGLT1	Sodium–glucose cotransporter 1
SGLT2	Sodium–glucose cotransporter 2
SGLT2is	Sodium–glucose cotransporter 2 inhibitors
